# *Trans*-Acting RNA–RNA Interactions in Segmented RNA Viruses

**DOI:** 10.3390/v11080751

**Published:** 2019-08-14

**Authors:** Laura R. Newburn, K. Andrew White

**Affiliations:** Department of Biology, York University, Toronto, ON M3J 1P3, Canada

**Keywords:** RNA structure, RNA–RNA interactions, RNA virus, RNA packaging, segmented virus, influenza virus, bluetongue virus, reovirus, red clover necrotic mosaic virus

## Abstract

RNA viruses represent a large and important group of pathogens that infect a broad range of hosts. Segmented RNA viruses are a subclass of this group that encode their genomes in two or more molecules and package all of their RNA segments in a single virus particle. These divided genomes come in different forms, including double-stranded RNA, coding-sense single-stranded RNA, and noncoding single-stranded RNA. Genera that possess these genome types include, respectively, *Orbivirus* (e.g., Bluetongue virus), *Dianthovirus* (e.g., Red clover necrotic mosaic virus) and *Alphainfluenzavirus* (e.g., Influenza A virus). Despite their distinct genomic features and diverse host ranges (i.e., animals, plants, and humans, respectively) each of these viruses uses *trans*-acting RNA–RNA interactions (tRRIs) to facilitate co-packaging of their segmented genome. The tRRIs occur between different viral genome segments and direct the selective packaging of a complete genome complement. Here we explore the current state of understanding of tRRI-mediated co-packaging in the abovementioned viruses and examine other known and potential functions for this class of RNA–RNA interaction.

## 1. Introduction

RNA viruses are important pathogens that infect a wide variety of hosts. The RNA genomes of these viruses can exist in different forms [1]. Those that are single-stranded (ss) can be either positive-sense [(+) RNA, i.e., messenger-sensed] or negative-sense [(−)RNA, i.e., complementary to the coding strand]. Another class of RNA virus includes those that utilize double-stranded (ds) RNA for their genomes. All of these viruses can be further categorized by the number of genome segments they possess. Those with a single genome segment are referred to as non-segmented, while those with a genome divided into two or more segments are defined according to the scheme by which their genomes are packaged. Viruses that package all of their genome segments into a single particle are called segmented viruses, whereas those that package their genome segments into two or more particles are referred to as multipartite viruses [2,3].

Segmented RNA viruses include all three of the abovementioned RNA genome types and examples of those with (−)RNA, dsRNA, and (+)RNA genomes include, respectively, alphainfluenzaviruses, orbiviruses, and dianthoviruses. Although these viruses benefit from their ability to reassort genome segments, which allows for replacement of defective segments and rapid evolution, there are also drawbacks associated with partitioning a genome [4]. For instance, these viruses face the task of packaging two or more genome segments into a single virion. This represents a daunting challenge for viruses like alphainfluenzaviruses, which contain eight genome segments, [5]. Clearly, such events must involve some form of communication between the different segments to achieve incorporation of one copy of each into a single particle. The required crosstalk could be mediated by viral or cellular factors, such as proteins. Alternatively, direct interactions between genome components, i.e., intermolecular or *trans*-acting RNA–RNA interactions (tRRIs), could also facilitate co-packaging. Indeed, the latter strategy appears to be involved in this process for a number of segmented RNA viruses [6,7,8]. Additionally, there is at least one clear example where the function of a tRRI extends beyond packaging [8]. Accordingly, the potential for this class of interaction to mediate different viral processes may not yet be fully appreciated. Herein, we examine and discuss the roles of tRRIs in the reproductive cycles of certain segmented RNA viruses that possess (−)RNA, dsRNA, and (+)RNA genomes.

## 2. Alphainfluenzaviruses

**Figure 1:**
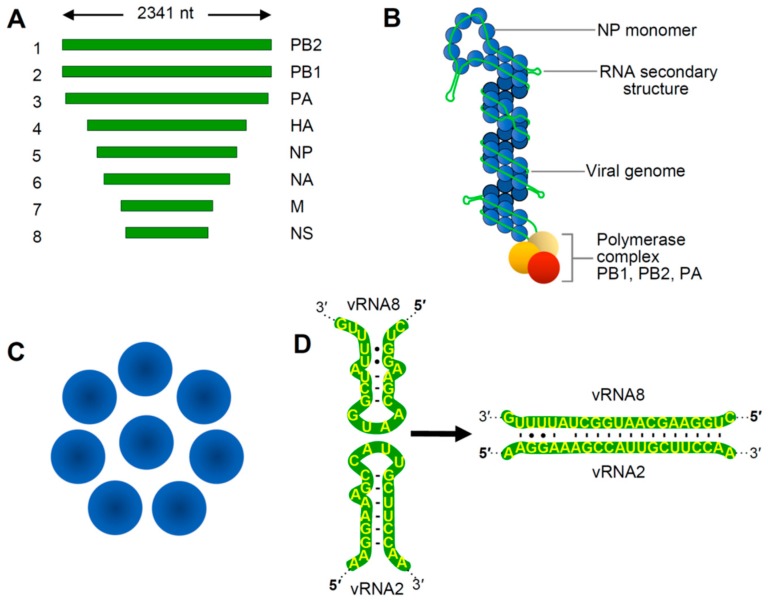
Genome-related structures for Influenza A virus. (**A**) Linear representation of influenza A virus (IAV) genome segments, labeled 1 to 8 (left), along with corresponding proteins encoded by each (right). (**B**) Schematic representation of a generic IAV vRNP complex with relevant structural features labelled. Not to scale. (**C**) Axial view of 7+1 arrangement of vRNPs depicting their parallel alignment inside IAV particles. (**D**) Predicted RNA secondary structures of interacting RNA regions in vRNP 8 and vRNP 2 (left) and their in trans RNA–RNA interaction (right) that mediates vRNP association [9]. The local RNA secondary structures (left) are predicted to extend from their respective vRNPs (as shown in B, labeled, RNA secondary structure), thereby facilitating nucleation the interaction.

The genus *Alphainfluenzavirus* belongs to the family *Orthomyxoviridae* and includes important pathogens of animals and humans [5]. Influenza A virus (IAV), the type genus of the family, has a (−)RNA genome composed of eight linear segments that range from 2.3 to 0.9 kb in length (Figure 1A). Viral genome replication occurs in the nucleus via a (+)RNA antigenome intermediate, and progeny (−)RNA genomes generated are transported as viral ribonucleoprotein (vRNP) complexes to the cytoplasm where they bud from the plasma membrane [5]. Within virions, the terminal regions of each segment are associated with RNA replication and transcription proteins (PB2, PA and PB1, the latter subunit being the RNA-dependent RNA polymerase or RdRp). The remaining portions of the genomes are coated with nucleocapsid protein (NP) (Figure 1B). Eight different vRNPs are encased by a matrix that is then covered by an outer lipid envelope to create a complete IAV virion.

Portions of the terminal regions of each genome segment are complementary and base paired, and RNA regions located more internally interact with NP to form a double helical structure (Figure 1B). NP-bound regions were originally believed to be uniformly coated, with NP associating with the viral RNA in a consistent manner. However, recent studies have revealed that NP, which interacts with ~12 nt long segments of sequence, is spread unevenly on viral RNA, exhibiting regions of high and low density [10,11,12]. This unequal distribution is likely dictated primarily by RNA sequences and/or structures that influence NP binding, however the specific structural features guiding the differences in concentration remain to be defined. It is also possible that NP binding may be able to remodel or direct the folding of RNA secondary structure, which could, in turn, influence accessibility for tRRI formation. Additionally, the stage at which NP subunits engage progeny genomes might influence the outcome of RNA–NP interactions, because RNA structures presented co-transcriptionally would differ from those in completely folded genomes. Regardless of the nature of vRNA assembly, RNA coinciding with areas of low NP occupation are predicted to be less restricted and able to form interactions with proteins or other RNAs. This feature is relevant because the eight vRNPs are organized in a conserved 7+1 configuration within virus particles [13,14], an arrangement that is anticipated to require communication between the discrete vRNPs (Figure 1C). Accordingly, NP-free regions of RNA in vRNPs could mediate such crosstalk.

IAV packaging appears to be a selective process [15,16] and *cis*-acting packaging signals have been identified proximal to the terminal regions of different genomic RNA segments [17,18]. Interestingly, some RNA sequences are important for the general selection of segments for packaging, while others ensure incorporation of a complete ensemble, or bundle, of eight unique segments [19,20]. As mentioned above, NP-free regions of RNA represent excellent candidates to mediate the bundling process, because they could confer the specificity required to distinguish between individual segments and mediate the unique interactions needed to form a 7+1 vRNP bundle (Figure 1B,C). In this respect, experimental evidence from different approaches exists that supports a tRRI-mediated mechanism for bundling. In vitro RNA–RNA electrophoretic mobility shift assays (EMSAs) were used to identify regions responsible for interactions between IAV genome segments, and the results suggested the existence of a network of interactions involving different RNA segments [21,22]. Importantly, one of the predicted base pairing interactions, which forms between the PB1 and NS segments, was shown to be important for packaging in virus infections by disrupting and then restoring the interaction with alternative base pairs (i.e., compensatory mutations) [9]. The local sequences in the two participating segments in this interaction were both predicted to form hairpins, with complementary loop sequences that could nucleate pairing via a kissing loop interaction (Figure 1D). This observation reinforces the idea that examining the local structural context should be factored into tRRI analyses, because it can greatly influence interaction efficiency and may guide temporal aspects of interactive pathways. The structure–function correlation provided by the compensatory mutational analysis in this study represented the earliest compelling evidence for tRRIs directing the selection and incorporation of specific vRNPs into bundles.

**Figure 2:**
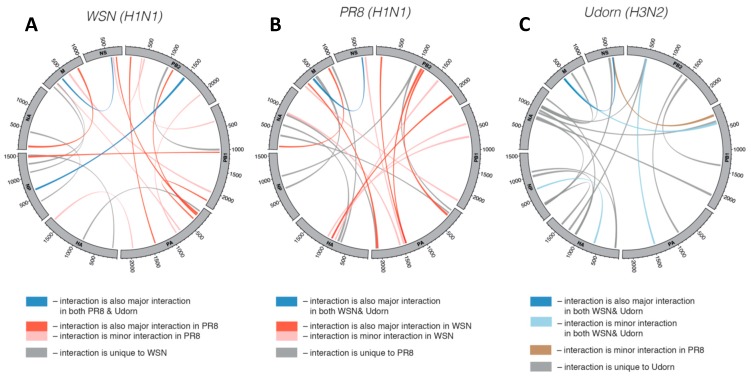
Summary of positions in IAV genome segments involved in inter-segment interactions in different IAV strains [24]. The eight segments are shown aligned in a circle with inter-segment interactions represented by curved coloured lines. See legends for correlations of interactions between IAV strains. (**A**) WSN (H1N1). (**B**) PR8 (H1N1). (**C**) Udorn (H3N2).

Further support for interactions between vRNPs has come from electron tomographic analyses of bundles that indicated connections between vRNPs, which may be tRRIs [13,14,23]. Recently, another study employing RNA–RNA crosslinking of viral RNAs within intact IAV particles (in virio) uncovered numerous tRRIs interactions dispersed widely throughout genome segments (Figure 2A) [24]. In the WSN (H1N1) strain, the top 10% of the interactions identified contained from 10 to 45 bp (averaging ~22 bp), however most (~68%) contained two or more interruptions (e.g., mismatches, bulges, or internal loops) within the base paired segments. The interactive genomic network also revealed multiple interactions between pairs of segments, as well as individual segments interacting with several different segments. This type of multidimensional network would have the interactive complexity required to direct arrangement of the 7+1 configuration. Moreover, some sequences were able to pair with multiple partner sequences, indicating mutual exclusivity for some interactions (i.e., only one of the possible interactions could occur at a time). When the interactive networks of different strains were compared, distinct sets of tRRIs were observed, revealing that networks can be unique in species subtypes (Figure 2A–C). Notably, this study also included compensatory mutational analysis of an interaction between strain-specific PB1 and NA segments that demonstrated its role in directing preferential co-segregation under competitive conditions [24]. Accordingly, tRRIs can influence genetic reassortment and represent an important factor to consider when calculating the probability of reassortment between strains. It follows that such analysis would be helpful for risk assessments of the emergence of new IAV strains [24].

Redundancy of tRRIs could provide a level of interactive fluidity that would safeguard against impairment due to loss of any one interaction, as supported experimentally [25]. This feature could also provide a safe context to explore new interactions or modify existing ones without loss of function. The ability of vRNPs to form and maintain tRRIs within a given 7+1 bundle would also be influenced by the distance between interacting vRNPs and the allowable extension lengths of the partner RNA sequences. These and other factors would operate to guide and constrain bundle architecture. An interactive network would also dictate how bundles are assembled spatially and temporally, a process that is proposed to be hierarchical [26,27]. However, based on the large number of inter-segment interactions observed [24], there may be a level of flexibility in terms of the pathway utilized to attain the final 7+1 arrangement.

Electron tomography has provided insights into the arrangement of different vRNPs within bundles [14,23]. Based on differences in length, vRNPs could be roughly assigned to positions within the 7+1 configuration. In one study, the central vRNP was consistently long, suggesting its cognate genome was one of segments 1 through 4 [23]. The remaining three long vRNAs were never found aligned consecutively in the peripheral ring. This finding was consistent with the results of a second study that assigned vRNP 4 as the central hub, with two of the remaining three long vRNPs positioned sequentially in the outer ring and the third positioned across the center from them [14]. Further refinement, by including in vitro interaction data, guided the assignment of vRNP 5 next to 8, and vRNP 6 next to 7, in the outer ring. It remains to be determined whether a specific configuration of vRNPs is formed consistently within and between strains, or if alternative arrangements are functionally acceptable. Regardless of the configurational flexibility of the final product(s), the structure of such complexes will undoubtedly be guided by tRRIs.

The cellular location and order of addition of vRNPs during the formation of 7+1 bundles remain largely unknown, and stages in assembly could occur before nuclear export and/or at different points during cytoplasmic transport to the plasma membrane [28,29,30]. The recent report of vRNPs present in cytoplasmic inclusions that possess properties of liquid organelles indicates that the vRNPs may be actively sequestered to facilitate tRRIs [31]. Finally, although tRRIs are clearly involved in genome packaging, they may also be important for maintaining bundles after virus entry, by facilitating delivery of a complete genome complement to the nucleus [29].

## 3. Orbiviruses

*Reoviridae* comprises a large family of dsRNA viruses that collectively infect a broad range of hosts, including fungi, plants, vertebrates, and invertebrates. Bluetongue virus (BTV), in the subfamily *Sedoreovirinae*, infects domestic and wild ruminants and is the type species of the genus *Orbivirus* [32]. BTV is composed of ten linear dsRNA segments, S1 through S10, and is an important model system for understanding copackaging of viral RNAs in this virus family [33]. Genome replication of BTV is linked to virion assembly and occurs in the cytoplasm within inclusion bodies [32]. Following cell entry, viral RdRps within the capsid core transcribe the dsRNA genomes into mRNAs. The mRNAs are released into the cytoplasm where they are translated into viral proteins. Some of these messages ultimately become the coding stands of progeny dsRNA genomes. Conversion of viral mRNAs into dsRNA genomes occurs during virion assembly. The mRNAs are recruited into capsids along with their associated viral polymerases, which catalyzes the synthesis of complementary strands. This assembly process requires that one copy of each of the ten different viral mRNAs be recruited into the capsid. Current data indicate that this co-segregation of mRNAs involves tRRIs between these viral messages [33].

**Figure 3:**
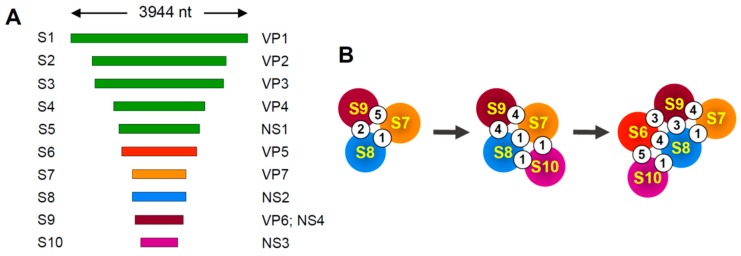
Genome-related structures for Bluetongue virus. (**A**) Linear representation of the BTV genome segments, labeled S1 to S10 (left), along with corresponding proteins encoded by each (right). Smaller segments are individually color-coded. (**B**) Schematic representation depicting the number of predicted interactions (white circles) occurring between different viral mRNAs (color-coded circles) during early RNA–RNA complex assembly in BTV [37].

BTV genome segments range from 3.95 to 0.8 kb in length and share conserved complementary termini [32] (Figure 3A). The 5′-termini of the coding strands in segment duplexes are capped, due to their derivation from the transcribed pool of viral mRNAs. Evidence for the involvement of tRRIs in BTV packaging originated from studies using a cell-free in vitro assembly system, RNA–RNA interaction assays, and an inhibitory oligonucleotide assay [34,35,36]. Systematic analyses of the contribution of different viral mRNAs to tRRI-mediated RNA complex formation and virus viability revealed a critical role for some of the smaller mRNAs corresponding to segments S7 through S10. This led to the hypothesis that packaging initiates with interactions between smaller viral mRNAs, with the subsequent addition of medium and larger ones [33].

Recently, a computational approach was used to predict a sequential assembly pathway to describe mRNA complex formation for segments S6 through S10 (Figure 3B) [37]. Briefly, the approach involved first identifying predicted ssRNA regions in each mRNA and then assessing potential tRRIs between these regions. This process was then repeated successively, with each iteration reassessing the effects of the newest interactions on prior intra- and intersegment interactions and their influence on the potential to form new interactions. The results yielded a pathway for segment assembly that involved a dynamic network of interactions (Figure 3B), with the lengths of interacting regions ranging from 9 to 18 base pairs and free energies spanning −16 to 27 kcal/mol. Interestingly, transitions from one stage to the next were predicted to involve both the addition of new interactions, as well as the loss of existing interactions [37]. The latter observation highlights the possible involvement of transient interactions during complex assembly. The importance of five interactions between S10 and S6 mRNAs that were predicted to be involved in the formation of final complex (Figure 3B) was confirmed by analyses of tRRI-disrupting mutants in interaction assays and virus infections. Interestingly, while the loss of function of one of the mRNA S10-S6 interactions caused by substitutions could be recovered by compensatory substitutions, the same was not true for a second interaction that was tested. The latter result suggests that localized changes can have global effects in a segment that lead to interference with other parts of the interactive network [37]. Additional functionally relevant BTV segment interactions have also been reported. Specifically, tRRIs between mRNAs for S5&S10 (involving 7 bp) and S1&S7 (8 bps) were identified and confirmed to be functionally important by compensatory mutational analyses using both in vitro binding and virus infectivity assays [38].

Collectively, these results provide a solid foundation for identifying additional BTV interactions and exploring the assembly pathways involved. Future studies will need to integrate all of the existing validated tRRIs into a unified model. Currently, most of the larger segments have not been analyzed extensively, thus their proposed roles in later stages of tRRI network assembly warrant further investigation. Also, it remains to be determined whether accurate assembly requires a tRRI network that includes all ten segments and, if so, how is a complete ensemble of ten “sensed” by the virus.

The genus *Rotavirus* is in the same *Sedoreovirinae* subfamily as orbiviruses, but these viruses contain eleven dsRNA genome segments. Similar to BTV, in vitro studies on rotaviruses (RVs) support the involvement of mRNA tRRI networks in segment packaging [39,40], however these tRRIs still require functional validation in virus infections. Interestingly, in vitro analysis of RV segment interactions revealed that virally-encoded NSP2, an RNA-binding protein, is able to remodel RNA segments in a manner that enhances tRRIs [40]. This finding may be functionally relevant, as NSP2 associates with the cytoplasmic inclusion bodies that are the sites of genome replication and packaging in infected cells [41]. In vitro, NSP2 binding to S11 RNA resulted in structural reorganization that led to the uncovering of ssRNA regions that facilitated inter-segment interactions. This RNA chaperone activity was proposed to be mediated by the high affinity of NSP2 for ssRNA, because a low affinity mutant was less effective. Additionally, NSP2 possesses helix-unwinding activities and is capable of efficient intramolecular RNA helix disruption, a property that could unmask sequences involved in tRRIs [42]. It has also been suggested that NSP2 could assist in preventing “unchaperoned” tRRIs between viral mRNAs during infections, which could be inhibitory to their translational function [43]. VP6 of BTV, which possesses RNA-binding activity, is implicated in facilitating packaging, but does not appear to be involved in mediating tRRIs in the assembly process [44]. Instead, other viral or host proteins could conceivably be involved in tRRI formation in orbiviruses.

## 4. Dianthoviruses

**Figure 4:**
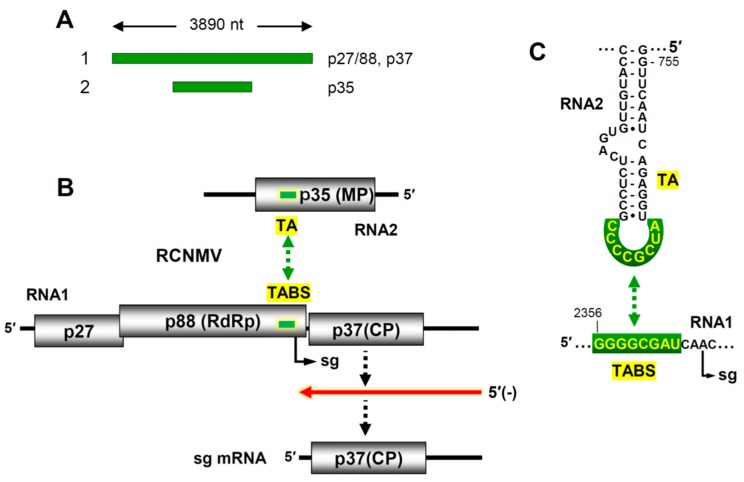
Genome-related structures for Red clover necrotic mosaic virus. (**A**) Linear representation of the RCNMV genome segments, labeled 1 and 2 (left), along with corresponding proteins encoded by each (right). (**B**) Schematic representation of RCNMV RNA1 and RNA2 and their encoded proteins (boxes). The relative location of the TA and TABS in each genome segment is indicated by a dark green bar. Sg mRNA transcription occurs via production of a sg mRNA-sized (−)RNA intermediate (red), that is then used as a template to transcribe the sg mRNA. (**C**) Depiction of the sequences (highlighted in green) that are involved in the TA–TABS tRRI between RCNMV RNA1 and RNA2 that activates sg mRNA transcription [47].

*Tombusviridae* is a large plant virus family whose members contain (+) RNA genomes [45]. All members of this family are non-segmented, except for those in the genus *Dianthovirus*. Red clover necrotic mosaic virus (RCNMV) is the best characterized species in this genus [8]. Its genome is composed of two linear (+) RNA segments with lengths of 3.9 and 1.45 kb that are copackaged into a 36 nm spherical capsid (Figure 4A). The larger RNA1 segment encodes an accessory replication protein (p27), its -1 frameshift product (p88) the viral RdRp, and the lone capsid subunit, p37 (Figure 4B) [46]. RNA2 codes for a single protein, p35, that mediates virus cell-to-cell movement. The viral RdRp is responsible for replication of both genome segments, which occurs via (-)RNA intermediates, and transcription of a subgenomic (sg) mRNA from RNA1. Transcription of the sg mRNA allows for expression of the capsid protein (CP), which is translationally silent in the context of RNA1 (Figure 4B).

Although RNA1 is capable of genome replication on its own, the transcription of the sg mRNA during infections requires the presence of both RNA1 and RNA2, signifying a communication requirement between the viral genome segments [48]. Further analysis led to the discovery that a tRRI between RNA1 and RNA2 was necessary for activation of sg mRNA transcription [47]. The interaction, confirmed with compensatory mutations, occurs between a sequence located just upstream from the sg mRNA initiation site in RNA1 and a complementary sequence in RNA2 positioned within the coding region of the p35 movement protein (Figure 4B). The interaction creates an RNA-based attenuation structure that causes premature termination of the RdRp during (-)RNA synthesis of RNA1, resulting in a truncated sg mRNA-sized (-)RNA that is used as a template to transcribe the sg mRNA (Figure 4B).

In RNA2, the inter-genomic interaction is mediated by an 8 nt long interacting sequence that is present in the terminal loop of a stem-loop structure, termed the *trans*-activator (TA). Base pairing of this TA loop with its complementary partner sequence in RNA1, the *trans*-activator binding site (TABS), forms a higher-order bimolecular RNA structure that halts progression of the RdRp (Figure 4C). Although the TA has been modelled in complex with the 8 nt TABS sequence [49], the overall tRRI structure that blocks the RdRp is unknown. Whether acting independently, or with the assistance of an associated protein factor, this tRRI is critical for activation of sg mRNA transcription and subsequent CP production. Notably, activation occurs optimally later in infections when the viral RNA levels are high, which coordinates the production of CP with ample levels of the two viral genome segments [47].

The same TA–TABS interaction is also proposed to serve a second major function that is akin to the roles played by tRRIs in IAV and orbivirus infections; i.e., mediating co-packaging of viral RNA segments via direct tethering [47]. However, although small RNA fragments containing TA and TABS are able to interact directly in vitro [49], comparable testing with the full-length RNA1 and RNA2 was unsuccessful [50]. This hints that a viral or host factor may be required to either mediate or/and stabilize the tRRI between the genome segments, as suggested for rotaviruses. It is also not known if other tRRIs exist between the two genome segments that might enhance the efficiency of co-packaging or regulate some other viral process.

Remarkably, the TA stem-loop also has additional activities. It is both a *cis*-acting RNA replication element necessary for efficient replication of RNA2 [51] and a primary packaging signal that interacts preferentially with CP [52,53]. This latter property implies that RNA1 may not be packaged efficiently if not tethered to RNA2. Consistent with this notion, examination of the contents of particles from wild-type infections found either one copy each of RNA1 and RNA2 or four copies of RNA2 [50]. The existence of RNA2-only-containing particles suggests that tRRIs, allowing for RNA2-RNA2 complex formation, may also exist.

Collectively, the TA represents an extraordinary multifunctional RNA element that is involved in at least four distinct viral processes. Its roles in sg mRNA transcription of the CP message, co-packaging of RNA segments, and selective genome encapsidation are all linked to tRRI-mediated particle assembly, whereas its *cis*-acting function in RNA2 genome replication is distinct. Further studies will be required to address how each of these different functions are regulated and coordinated during infections.

## 5. Conclusions and Perspective

Our sampling of three different types of segmented RNA viruses has revealed a common role for tRRIs—the selective co-packaging of genome segments. Many structural features of the complementary segments can influence such interactions, including sequence composition, length, and position. Equally important are the higher-order structural contexts of these RNA sequences, which influence their accessibility. Add to this the possibility of multiple tRRIs between a given pair of segments, and other considerations such as cooperativity and the hierarchical order of formation of these tRRIs come into play. An additional layer of complexity can be added if functionally relevant RNA remodelling occurs, mediated by either proteins or tRRIs themselves. Also relevant at different structural levels is the possibility of RNA modifications, such as N6-methyladenosine (m6A), which can affect both RNA-protein and RNA–RNA interactions [54]. Finally, it cannot be precluded that non-canonical types of RNA interactions may also be involved in forming tRRIs. Thus, many factors need to be considered collectively when investigating the structure and function of these interactions.

A variety of approaches including computational, biochemical, genetic, and cellular techniques have been used to identify and study the functions of viral tRRIs. The initial identification of putative interacting genome segments and corresponding complementary sequences involved computer-assisted RNA structural analyses [55], RNA–RNA EMSAs, and/or RNA–RNA crosslinking techniques. In several cases, tRRI candidates were verified to be functionally relevant in infections using the gold standard for such validations, compensatory mutational analysis [9,24,37,38,47]; however, many still remain to be confirmed experimentally. Unfortunately, applying this approach successfully can be challenging if interactions exist in coding regions, which restricts possible substitutions, or if there are redundant interactions, which could mask function. Fluorescence in situ hybridization (FISH) has been effective for visualizing different viral RNAs and assessing colocalization in cells [28,29], however it does not provide sufficient resolution to assign direct contact. Nonetheless, probable sets of interactions leading to IAV vRNP assembly have been generated by combining FISH-derived spatial proximity data with dynamic programming [30]. Results from different image-based analyses have been very informative, however full comprehension of the spatial and temporal aspects of assembly will likely require the development of novel technical approaches.

The tRRIs that form in different viruses are predicted to be disrupted at different stages after assortment. In BTV, inter-mRNA links are presumably lost when the messages are converted to dsRNA genomes during virion assembly, making them relatively transient. In contrast, tRRIs in IAV are maintained in virions and may even persist following fusion-mediated delivery into the cytosol of newly infected cells, possibly mediating codelivery of vRNPs to the nucleus [29]. For RCNMV, it is unclear if the TA–TABS interaction remains intact in virions or is still present after disassembly. Regardless, for IAV and RCNMV, disruption of tRRIs are projected to occur, respectively, during the replication and translation of their genomes. Such stability and reversibility needed at different stages of infections could be provided by collections of less stable tRRIs [24,37,38], a feature that may be further enforced by the location of many of them in coding regions (often both partner sequences), which markedly limits contiguous sequence pairing.

The presence of tRRIs in coding regions is also relevant to experiments that assess virus protein function by modifying its corresponding coding sequence, as substitutions or deletions can concomitantly disrupt network connections. The same knowledge may also be pertinent to certain applications, such as optimizing the design of expression vectors by integrating appropriate partner tRRIs with introduced foreign genes. As one would expect, tRRIs also influence the efficiently of reassortment, as demonstrated experimentally for IAV [24]. Likewise, tRRI compatibility is predicted to influence inter-strain segment exchanges in RCNMV and BTV. The minimum threshold that would allow reassortment to occur and how quickly a resulting “weak” reassortant could “evolve” more stable interaction(s) are questions of interest. For IAV, it is also possible that compensation for tRRI deficiencies could be restored via changes in NP. Other related unknowns also exist, such as the stability of tRRIs over time, and how networks morph during the course of single host infections, after extensive passaging, or in different hosts. Such investigations should provide clues as to the robustness and plasticity of tRRI networks and help to uncover factors that drive their evolution.

The study of tRRIs in segmented RNA viruses is a new and growing field in virology. Understanding the roles of this class of interaction will provide important basic knowledge on how selective packaging occurs in segmented RNA viruses. Information on tRRIs will also be useful in assessing the likelihood of new viral strains emerging via reassortment, and could guide the development of antiviral treatments based on inhibition of these interactions. Moreover, as observed for RCNMV, the possibility exists that the functions for tRRIs extend beyond their established role in genome assortment. As the field expands, it will be interesting to see if novel functional tRRIs are active in other types of segmented RNA viruses.
